# Conditioned Pain Modulation and Situational Pain Catastrophizing as Preoperative Predictors of Pain following Chest Wall Surgery: A Prospective Observational Cohort Study

**DOI:** 10.1371/journal.pone.0090185

**Published:** 2014-02-26

**Authors:** Kasper Grosen, Lene Vase, Hans K. Pilegaard, Mogens Pfeiffer-Jensen, Asbjørn M. Drewes

**Affiliations:** 1 The Department of Cardiothoracic and Vascular Surgery, Aarhus University Hospital, Aarhus, Denmark; 2 The Department of Psychology and Behavioral Sciences, Aarhus University, Aarhus, Denmark; 3 The Danish Pain Research Center, Aarhus University, Aarhus, Denmark; 4 The Department of Rheumatology, Aarhus University Hospital, Aarhus, Denmark; 5 Mech-Sense, Department of Gastroenterology and Hepatology, Aalborg University Hospital, Aalborg, Denmark; 6 Center for Sensory-Motor Interaction (SMI), Department of Health Science and Technology, Aalborg University, Aalborg, Denmark; University of Washington, United States of America

## Abstract

**Background:**

Variability in patients' postoperative pain experience and response to treatment challenges effective pain management. Variability in pain reflects individual differences in inhibitory pain modulation and psychological sensitivity, which in turn may be clinically relevant for the disposition to acquire pain. The aim of this study was to investigate the effects of conditioned pain modulation and situational pain catastrophizing on postoperative pain and pain persistency.

**Methods:**

Preoperatively, 42 healthy males undergoing funnel chest surgery completed the Spielberger's State-Trait Anxiety Inventory and Beck's Depression Inventory before undergoing a sequential conditioned pain modulation paradigm. Subsequently, the Pain Catastrophizing Scale was introduced and patients were instructed to reference the conditioning pain while answering. Ratings of movement-evoked pain and consumption of morphine equivalents were obtained during postoperative days 2–5. Pain was reevaluated at six months postoperatively.

**Results:**

Patients reporting persistent pain at six months follow-up (n = 15) were not significantly different from pain-free patients (n = 16) concerning preoperative conditioned pain modulation response (*Z* = 1.0, *P* = 0.3) or level of catastrophizing (*Z* = 0.4, *P* = 1.0). In the acute postoperative phase, situational pain catastrophizing predicted movement-evoked pain, independently of anxiety and depression (*β* = 1.0, *P* = 0.007) whereas conditioned pain modulation predicted morphine consumption (*β* = −0.005, *P* = 0.001).

**Conclusions:**

Preoperative conditioned pain modulation and situational pain catastrophizing were not associated with the development of persistent postoperative pain following funnel chest repair. Secondary outcome analyses indicated that conditioned pain modulation predicted morphine consumption and situational pain catastrophizing predicted movement-evoked pain intensity in the acute postoperative phase. These findings may have important implications for developing strategies to treat or prevent acute postoperative pain in selected patients. Pain may be predicted and the malfunctioning pain inhibition mechanism as tested with CPM may be treated with suitable drugs augmenting descending inhibition.

## Introduction

Pain is an expected part of surgical recovery but effective pain management remains challenging [1]–[4]. The high variability in postoperative pain experience and analgesic treatment response between patients is part of the challenge [5], [6]. It has been suggested that variability in the patients' ability to modulate pain (endogenous pain modulation) may be clinically relevant for the variability of and disposition to acquire pain [7]–[12].

Endogenous pain modulation has been experimentally investigated in humans via conditioned pain modulation (CPM) paradigms, during which central inhibition of a painful stimulus is induced by applying a second painful conditioning stimulus to a remote body region (i.e., counter-irritation) [8], [13]. Deficits in preoperative CPM have been found to be associated with chronic pain following thoracotomy, i.e., the chance of a patient who reported a decrease in heat pain intensity scores from 50/100 at baseline to 40/100 during hot water hand immersion to develop chronic postoperative pain was about one-half that of a patient who reported unchanged scores [14]. Inhibitory pain modulation and pain intensity are strongly influenced by psychological features [10]. Pain catastrophizing, defined as an exaggerated negative orientation toward pain stimuli and pain experience has emerged as a robust psychological predictor of clinical pain outcomes [15], [16]. Higher levels of preoperative pain catastrophizing have been associated with higher postoperative pain ratings [17]–[20]. However, the majority of these studies have assessed *dispositional* catastrophizing (i.e., recall of catastrophizing thoughts during previous pain events), which probably measures different pain experiences than *situational* catastrophizing (i.e., catastrophizing thoughts measured directly after exposure to a noxious stimulation) [21]–[23]. In the literature, situational pain catastrophizing has shown more robust correlations with pain-related outcomes than dispositional measures of pain catastrophizing [16], [21]–[23]. Although it has been shown that catastrophizing in response to experimental heat pain accounted significantly for the variance in pain after cesarean section [20], there are no studies on the association between preoperative situational pain catastrophizing and postoperative pain in males undergoing thoracic surgery. Finally, considerable evidence suggests the importance of examining unique versus common contributions of pain catastrophizing and empirically and conceptually related variables, particularly anxiety and depression, to pain-related outcomes [24]–[26]. Nonetheless, previous research has inconsistently controlled for these negative affect constructs when assessing relationships between pain catastrophizing and pain-related outcomes in surgical patients. The mechanisms by which catastrophizing modulate pain are not completely understood [10]. Previous studies have shown that CPM may be influenced by catastrophizing, but evidence is equivocal [27]–[31] and the association between catastrophizing and CPM remain unclear.

To the best of our knowledge, no studies have yet combined preoperative assessment of CPM with measurements of cognitive and emotional processes in the prediction of postoperative pain. Such a study would add to our understanding of the causes underlying individual differences in pain and potentially enable identification of patients at high risk of developing persistent postoperative pain. In turn this may have important implications for developing multimodal pharmacological or psychological intervention strategies to treat or prevent postoperative pain in the future. We hypothesized that patients with a preoperative deficient endogenous pain inhibition capacity, expressed by less efficient CPM and/or high levels of situational pain-related catastrophic thinking, were more susceptible to postoperative pain and pain persistency.

## Materials and Methods

The current study was a prospective observational cohort study designed to investigate whether preoperative assessment of endogenous pain inhibition capacity can predict the course of postoperative pain in a consecutive cohort of patients undergoing surgical correction of funnel chest (pectus excavatum). Funnel chest is the most common congenital deformity of the anterior chest wall and the male/female ratio is approximately 6/1 for patients undergoing surgical correction of funnel chest at our institution. The deformity is primarily treated to achieve anatomical correction and thus avoid cosmetic and psychological inconveniences for the patient. Patients undergoing surgical funnel chest repair are generally healthy pain-free opioid naïve adolescents. However, they are at high risk of experiencing moderate to severe acute postoperative pain.

**Figure 1:**
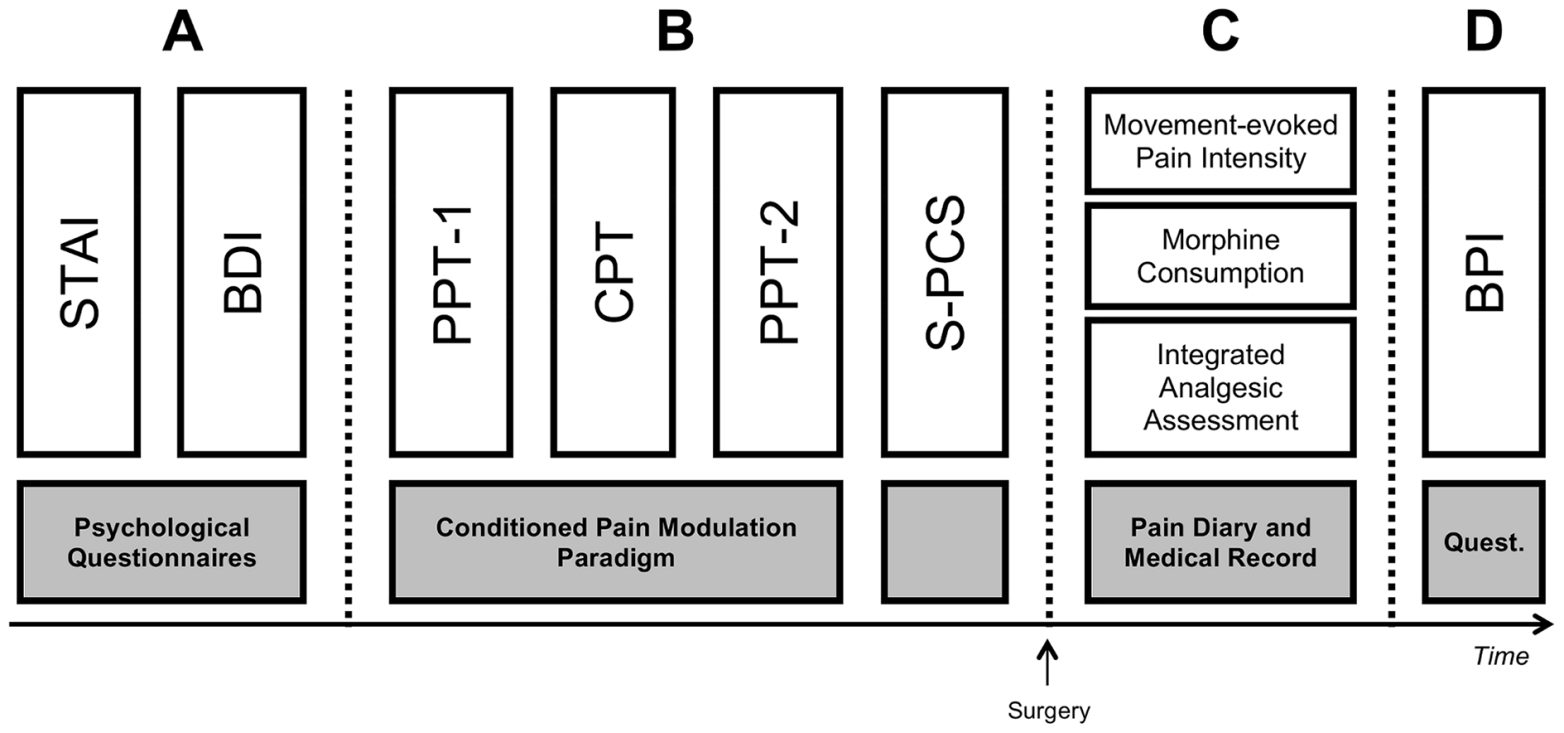
Schematic illustration of the study protocol. (**A**) The day before scheduled surgery patients completed the Spielberger's State-Trait Anxiety Inventory (STAI) and Beck's Depression Inventory (BDI); (**B**) Then patients underwent a conditioned pain modulation paradigm in which a baseline pressure pain threshold was measured at the quadriceps muscle followed by a conditioning painful stimulus induced by a cold pressor test (CPT) (i.e., hand immersion in an ice water bath for 120 seconds). After 120 seconds of hand immersion (or upon spontaneous hand removal) the pressure pain threshold at the quadriceps muscle was reassessed. The Situational Pain Catastrophizing Scale (S-PCS) was re-administered within five minutes after cold pressor test and patients were instructed to reference the cold pressor pain while answering; (**C**) From postoperative days 2–5 pain-related outcomes were assessed, including postoperative movement-evoked pain intensity, morphine consumption, and an integrated analgesic assessment score based on the aforementioned; (**D**) Persistent postoperative pain was assessed according to responses to the Brief Pain Inventory – Short Form (BPI) at six months.

A schematic illustration of the study protocol is depicted in Fig. 1. The study consisted of a preoperative session in which we conducted dynamic quantitative sensory testing (CPM) and introduced a number of psychological questionnaires, including measures of pain catastrophizing, anxiety, and depression. CPM and pain catastrophizing were evaluated as equal candidate predictors of a range of clinical postoperative pain measures obtained over a 6-month period; the primary endpoint was persistent postoperative pain at six months. Anxiety and depression were considered as potential confounders of the relationship between pain catastrophizing and pain-related outcomes. This is the first report from the study where we compare persistent postoperative pain development and the course of acute postoperative pain according to patients' preoperative CPM scores and levels of pain catastrophizing in response to experimental pain.

### Ethics Statement

The Regional Committee on Biomedical Research Ethics (M-20110064) and the Danish Data Protection Agency (J. no.: 2011–41–6061) approved the study. The study was registered in the clinicaltrials.gov database (Identifier: NCT01308385; Principal Investigator: K.G.; Date of Registration: March 3, 2011) and was conducted in accordance with the Declaration of Helsinki. Patients admitted to surgery received written information explaining the purpose of the study along with the appointment for surgery. Upon arrival at the department, the principal investigator (K.G.) restated the study purpose verbally to eligible patients. All participating patients provided written informed consent. Of note, the 15–17-year-old patients received special information adapted to their age and abilities, both in language and in content. To further aid decision-making, parents, next of kin, caretakers, or guardians who had parental rights and responsibilities of underage patients, received separate information in writing and also participated in the conversation about the study purpose and possible participation of the young. The Ethics Committee granted special dispensation for the underage patients to provide written informed consent autonomously.

### Setting and Patients

Patients were recruited consecutively from the Department of Cardiothoracic and Vascular Surgery, Aarhus University Hospital, Denmark, during a pre-determined one-year period (May 2011 to May 2012). Patients were prospectively enrolled according to the following inclusion criteria: (1) elective minimally invasive surgical correction of funnel chest (pectus excavatum); and (2) age ≥15 years. Exclusion criteria were: (1) previous thoracic surgical interventions; (2) presence of diseases affecting the central and/or peripheral nervous system; (3) presence of chronic pain conditions; (4) inability to speak and/or understand Danish; (5) inability to understand and participate in the experimental pain session; (6) presence of psychiatric disorders; (7) history of frostbite in the non-dominant upper limb; (8) presence of sores or cuts on non-dominant upper limb; (9) presence of cardiovascular disease; (10) history of fainting and/or seizures; (11) presence of fractures of the non-dominant upper limb; or (12) presence of Reynaud's phenomenon. Secondary exclusions included: (1) insensitivity to experimental cold pressor pain; (2) lack of epidural catheter placement; or (3) re-operation.

### Study Overview

All preoperative assessments were conducted by the principle investigator (K.G.), the day before scheduled surgery in a quiet room with a constant temperature of approximately 20–22°Celsius. Initially, patients were informed briefly about the purpose of the study, excluding any information about examination of CPM and the focus on pain catastrophizing, and asked to read and sign a consent form. Patients were then asked to fill in the Spielberger's State-Trait Anxiety Inventory (STAI) and Beck's Depression Inventory (BDI). Subsequently, a standardized written statement based on instructions published by Price *et al.* was used to explain the reporting of pain intensity and unpleasantness using an 11-point numerical rating scale (NRS), where 0 =  no pain/unpleasantness and 10 =  worst pain/unpleasantness imaginable [32]. Afterwards, simple bedside sensory testing was carried out approximately 10 cm distal to the papilla on both sides of the thorax corresponding to 5 cm above the (planned) surgical incisions. Sensory testing included, assessment of dysesthesia and allodynia by gently stroking the patient's skin with a 1 cm wide; soft brush (SenseLab^TM^ Brush-05, Somedic AB, Hörby, Sweden) and mechanical dynamic hyperalgesia using a single von Frey monofilament (Touch-Test sensory evaluator, 5.88/60 g, nominal bending force 588.2 mN, Semmes-Weinstein monofilament, Stoelting Co., IL, USA) as previously described by Stubhaug *et al.* [33]. Patients were then familiarized with the pain measures involved in a sequential CPM paradigm. All study equipment was presented to the patient, and introductory pressure algometry training measurements were carried out on the patient's dominant forearm. Patients were instructed how to immerse and maintain their hand in a stirred ice water bath and to verbally report the moment they started feeling pain during the ice water hand immersion (registered as cold pain detection threshold measured in seconds elapsed from baseline). Accordingly, the cold pain tolerance threshold was defined as the latency to intolerability in seconds (spontaneous hand removal from the ice water bath). Successively, the CPM response was assessed. First, a single baseline pain threshold was measured using pressure algometry at the quadriceps muscle. The conditioning painful stimulus was then induced using hand immersion in the ice water bath (cold pressor test). After 120 seconds of hand immersion (or upon spontaneous hand removal) the pressure pain threshold at the quadriceps muscle was immediately reassessed. Upon hand withdrawal, patients were asked to numerically rate the worst (maximum) pain intensity and unpleasantness associated with ice water hand immersion on a NRS. Within five minutes after hand removal, patients were instructed to reference the conditioning stimulation (i.e., cold pressor pain) while filling in a Situational Pain Catastrophizing Scale (S-PCS). During pain testing procedures patients were seated comfortably on a chair without armrests and with both feet placed on the floor to achieve an approximately 90° flexing of the knee. Furthermore, patients were asked to look away from the test area and either close their eyes or fix their gaze on the wall in front of them to obtain full concentration. The next morning patients underwent standardized anesthesia and surgery, and movement-evoked pain was assessed on five consecutive postoperative days. Upon discharge, a review of each patient's electronic medical chart was conducted and data on analgesic consumption was retrieved. Patients were assessed with regard to persistent postoperative pain at six months according to responses to a detailed e-mailed questionnaire, including the Brief Pain Inventory – Short Form (BPI).

### Preoperative Predictors

#### Conditioned Pain Modulation

Mechanical Pressure Pain Threshold – the Test Stimulus. Pressure algometry was performed at the patients' quadriceps muscle (rectus femoris) 10 cm above the patella on the same side as the dominant hand. A handheld digital pressure algometer with a probe size of 1 cm^2^ was used (Algometer, Somedic AB, Hörby, Sweden). The pressure was gradually increased with a rate of 30 kPa per second. Patients were instructed to press a button when the feeling of pressure changed into a sensation of pain. This in turn froze the assessment parameter, i.e., the pressure measured in kPa at the pressure pain threshold on the algometer's display and the value was instantaneously stored it in the internal memory of the apparatus. The algometer was calibrated between patients using a manufacturer-supplied weight equal to 100 kPa.

Cold Pressor Test – the Conditioning Stimulus. Patients were asked to place their non-dominant hand in a stirred ice water bath (1±1°Celsius) in a still position with their fingers spread for as long as possible. A cut-off time of 120 seconds was set for safety reasons. The CPM effect was evaluated by comparing the baseline pressure pain threshold with the pressure pain threshold following the cold pressor test. In order to control for individual variation in baseline measures, the proportion of difference in pressure pain thresholds from baseline was used rather than the raw difference, and expressed in percentages. This approach results in negative CPM scores for pain facilitation and positive CPM scores for pain inhibition. In other words a positive CPM score was considered indicative of effective endogenous pain modulation [13]. Patients were told that pressure algometry conducted at baseline would be repeated following the cold pressor test, but they were not informed about the specific purpose of the repeated testing or of the expected results. Similarly, patients and staff involved in the postoperative care and treatment were blinded to the results of the CPM assessment during the entire study.

#### The Situational Pain Catastrophizing Scale (S-PCS)

The tendency to engage in pain catastrophizing was assessed by means of the Danish version of the Pain Catastrophizing Scale (PCS) [34], [35]. Pain catastrophizing is characterized as the tendency to magnify the threat value of pain stimulus and to feel helpless in the context of pain, and by a relative inability to inhibit pain-related thoughts in anticipation of, during or following a painful encounter [15], [16]. The PCS measures thoughts and feelings when experiencing pain using the following instruction: “We are interested in the types of thoughts and feelings that you have when you are in pain”. The scale has 13 items and includes three subscales: “rumination”, “magnification”, and “helplessness”. PCS scores range from 0 to 52, with lower scores indicative of less catastrophizing. The Danish version of the PCS has previously shown a high reliability (Chronbach's Alpha: 0.96) [24]. We aimed at examining situational pain catastrophizing since this approach has produced more robust correlations with pain-related outcomes compared with dispositional measures of pain catastrophizing [16], [21]–[23]. However, the situational use of the PCS after the cold pressor test required the original instructions to be appropriately revised: “We are interested in the thoughts and feelings that you had during the painful cold water hand immersion you have just experienced” (S-PCS) [16], [21], [22].

### Potential Confounding Variables

Pain catastrophizing shares variance with negative affect constructs such as depression and anxiety. It is thus important to statistically control for depressive symptoms and level of anxiety when investigating relations between pain catastrophizing and pain-related outcomes (i.e., the uniqueness of the catastrophizing construct) [16].

#### The Spielberger's State-Trait Anxiety Inventory (STAI)

Anxiety was measured with the Danish version of the state part of the Spielberger's State-Trait Anxiety Inventory (STAI) for adults [36]. The state part of the STAI measures emotional, cognitive and behavioral aspects of anxiety using 20 items for assessing state anxiety. The Danish version of the STAI has previously shown a reasonable reliability (Chronbach's Alpha: 0.80) [24].

#### Beck's Depression Inventory (BDI)

Depression was assessed with the Danish version of Beck's Depression Inventory (second edition) (BDI) consisting of 21 items assessing psychological and physiological aspects of depression over the preceding two weeks [37]. Numerous studies have shown the BDI to be a reliable and valid measure of depressive symptoms [38], and the Danish version of the BDI has also previously shown a high reliability (Chronbach's Alpha: 0.94) [24], [39].

### Primary Outcome

#### Persistent Postoperative Pain

The primary outcome measure was the presence of persistent postoperative pain at six months, and the potentially predictive role of CPM on such pain. Persistent pain was assessed according to responses to the Brief Pain Inventory – Short Form (BPI) [40]. The introductory question of the BPI was revised to ask specifically about the experience of surgery-related pain during the past week. In brief, the BPI rates worst, average, current, and least pain using simple numeric rating scales (NRS) ranging from 0 to 10, where 0 =  no pain and 10 =  the worst pain imaginable. Average pain was chosen as the main pain intensity criterion, as this item has been considered a measure of the patient's subjective average experience of pain [41]. The reliability and validity of the BPI is well established and has become a widely used measurement tool for assessing clinical pain in cardiothoracic surgery [42]–[45].

### Secondary Postoperative Pain-related Outcomes

#### Subjective Movement-evoked Pain Intensity

Postoperative movement-evoked pain was self-reported by patients in a study-specific pain diary based on NRSs. It is recommended to use NRSs in adult clinical trials [46] and it is a validated tool to measure both intensity and unpleasantness of acute pain in children and adolescents [47], [48]. Furthermore, the pain assessment was standardized and maneuver-specific [49]. Accordingly, the same movement was used to evoke pain in all patients, i.e. rising from a supine position (patient lying flat on a bed) to an upright position (patient standing next to the bed). We chose movement-evoked pain as outcome because this measure is generally more severe in intensity than pain at rest [4], [49]. Moreover, the deliberate avoidance of pain-evoking movement (e.g., deep breathing, coughing, and ambulation) contributes to impairment of postoperative functional recovery [50] The pain diary contained comprehensive instructions and a sample page to assist in completion of the measures; the diary was filled in every evening between 20:00 and 22:00 from the day of surgery to postoperative day 5.

#### Consumption of Morphine Equivalents

Data on postoperative opioid use following epidural analgesia (i.e., over postoperative days 2–5), independent of route or mode of administration was extracted from the electronic medical records or pain diaries if the patient was discharged before postoperative day 5. Missing data on morphine consumption on postoperative days 4 and 5 were acceptably imputed using the fixed daily morphine dose prescribed at discharge. Doses of opioid other than morphine were converted to morphine equivalents using standard equianalgesic dose conversion ratios (i.e., 1.5 for oxycodone, 0.2 for tramadol, and 210 for fentanyl) and calculated as mg kg^−1^ day^−1^ [51], [52] The cumulated use of epidural analgesia (i.e., bupivacaine and morphine) may be largely dependent of epidural catheter placement and was thus only calculated as supplementary information.

#### Integrated Analgesic Assessment Score

It has been suggested that using pain scores and analgesic requirements as isolated variables may prevent identification of the total benefit provided by the analgesic regimen. Hence, an integrated analgesic assessment score was calculated as described by Silverman *et al*. [53], [54]. Movement-evoked pain intensity scores and morphine consumption for each patient were rank ordered, subtracted from the mean rank, expressed as a percentage difference from the mean rank and added together (i.e., pain rank score + morphine rank score) to yield an integrated analgesic assessment score (ranging from −200 to +200%; with the highest positive score indicating the most pain). Patients ranging from +100 to +200 had a high pain score requiring higher-than-average morphine, whereas patients with scores ranging from −200 to −100 had a low pain score requiring less-than-average morphine [54].

### Anesthesia, Surgery and Analgesia

#### Anesthesia

All patients followed a standardized anesthesia regimen. Prior to the induction of general anesthesia, an epidural catheter placed at the thoracic levels 4–6. With the patient sitting in an upright position, using local anesthesia and a paramedian approach, the catheter was placed using a hanging drop technique. The epidural block was activated and tested with an injection of 0.5% bupivacaine with simultaneous intravenous infusion of a 6% hydroxyethyl starch. General anesthesia was initiated with intravenous injection of fentanyl (0.05–0.1 mg), propofol (1.5–2.5 mg kg^−1^) and cisatracurium (0.1–0.15 mg kg^−1^).

#### Surgery

The surgical technique was standardized and has previously been described in detail [55]. In brief, one or more convex steel bars are inserted under the sternum through small bilateral incisions in the thoracic wall to achieve sternal elevation and improve cosmetic appearance. The same highly experienced thoracic surgeon (H.P.) performed all surgical procedures.

#### Analgesia

The aim of postoperative analgesia was effective respiratory function and physical activity. Postoperative analgesia was tailored to the patient's individual requirements (i.e., titration to pain intensity less than 3/10 at rest and less than 5/10 upon cough/movement). The standardized analgesic regimen combined epidural analgesia, and non-opioid and opioid analgesics as described below. This regimen has previously been linked to enhanced recovery in fast-track surgery [56].

- Epidural Analgesia
  The preoperative epidural blockade was reactivated during surgery or upon arrival in the Post Anesthesia Care Unit at the discretion of the attending anesthetist and subsequently provided continuously for an additional 48 hours. Epidural analgesia was provided with continuous infusion of 0.25% bupivacaine + morphine, 50 µg ml^−1^ for the first 24 hours and with 0.25% bupivacaine for the next 24 hours. Maximum epidural infusion rate was set at 10 ml h^−1^. Epidural analgesia was discontinued and the epidural catheter was removed at 9:00 on postoperative day 2 in all patients.
- Non-opioid Analgesia
  Non-opioid analgesic treatment was initiated in all patients with acetaminophen (4 g day^−1^) and ibuprofen (1200 mg day^−1^) from the day of surgery.
- Opioid Analgesia
  Opioid analgesic treatment was initiated with controlled-release morphine at 08:00 on postoperative day 1, and subsequently adjusted once daily based on the opioid requirements in the preceding 24 hours. Morphine was acceptably exchanged for oxycodone in case of intolerance and/or untreatable adverse effects related to morphine analgesia (i.e., urinary retention, respiratory depression, nausea and vomiting, and/or pruritus).
- Rescue Analgesia
  Intermittent epidural bolus injections were given if the patient was uncomfortable due to pain (2–4 ml bolus; lockout of 15–20 minutes). If the patient required additional boluses within the hour, the continuous basal infusion was either increased or the epidural catheter was retracted to optimize the spread of injected solutions and avoid fluctuations in the level of analgesia. Additionally, bolus intravenous injections of 2 mg of morphine followed by an upward titration in 1–2 mg increments were available as rescue analgesia throughout hospitalization.

### Study Size

The size of the study population was based on a conservative estimate for a predetermined 1-year study period. We wanted to avoid overlooking an actual difference in CPM effect between pain-free patients and patients developing persistent postoperative pain at six months. Power calculation was based on results from previous studies showing that 14% of patients undergoing similar chest wall surgery developed persistent postoperative pain at six months [57] and results from a study in chronic pain patients indicating that the increase in pressure pain thresholds after cold pressor test was reduced in patients with chronic pancreatitis (13%, SD  = 21%) when compared to healthy volunteers (39%, SD  = 22%) [58]. Accordingly, the statistical power of the study (β) was estimated at 86% under the following assumptions: (1) 50 patients would be included during the predetermined 1-year study period; (2) 7/50 patients (14%) would develop persistent postoperative pain at six months; (3) with a significance (α) level at 5%; and (4) with mean CPM responses at 39 in pain-free patients and 13 in patients developing persistent postoperative pain (common SD  = 21).

### Statistical Analyses

Stata/IC 12.1 for Mac (64-bit Intel) (StataCorp, College Station, TX, USA) was used for all statistical analyses. Two-tailed *P* values less than 0.05 were considered statistically significant unless stated otherwise. Categorical variables are presented as numbers (percentages); continuous variables with a normal distribution are described with mean ± standard deviation; whereas non-normally distributed data is described with median [interquartile range], unless stated otherwise. Non-normally distributed data was attempted log-transformed to normality.

A paired *t* test was used to test the null hypothesis that conditioning cold pressor pain had no effect on the response to pressure algometry. Spearman's rank correlation coefficients (ρ) were calculated as measures of dependence between preoperative, perioperative, and postoperative study variables including predictors, potential confounders and outcomes. Considering the small sample sizes and obviously skewed data, the Wilcoxon-Mann-Whitney rank sum test was used for primary analyses to compare the distributions of CPM responses, S-PCS, BDI, and STAI scores in pain-free patients and patients reporting persistent postoperative pain at six months.

For analyses of secondary outcomes, we performed three separate ordinary least squares (OLS) linear regressions of (i) movement-evoked pain; (ii) morphine consumption; and (iii) the integrated analgesic assessment score on each of the continuous covariates: conditioned pain modulation (CPM) and situational pain catastrophizing (S-PCS). State anxiety and depression were solely chosen to control the effect of pain catastrophizing on postoperative pain-related outcome measures. Following initial residual analyses it was found appropriate to log-transform the S-PCS score (Log[*S-PCS*]). Hence, for each of the three mentioned multiple regressions (i-iii), log[*S-PCS*] was entered in Model 1; CPM response in Model 2; and log[*S-PCS*], STAI score, and BDI score in Model 3.

Regression models were validated by visual inspection of diagnostic plots, including Q-Q plot of the residuals, residual versus predicted, residuals versus explanatory variable(s), and leverage versus squared residuals. If the leverage versus squared residuals plot revealed an observation with high leverage and large residuals, we performed regressions with and without this outlier to assess its potential impact. Additionally, we conducted robust regressions in which influential observations were automatically dropped, (i.e., observations with a Cook's distance greater than 1), and those with large absolute residuals were down-weighted. Model validation did not suggest severe assumption violation and the conclusions were broadly similar regardless of using model-based or robust standard errors.

In order to interpret and visualize the regression models, we calculated adjusted means (predictive margins) with 95% confidence intervals (95% CI) of the patient's postoperative movement-evoked pain intensity given different log-transformed values of preoperative situational pain catastrophizing and of postoperative morphine consumption given different values of preoperative CPM.

## Results

### Patients

**Figure 2:**
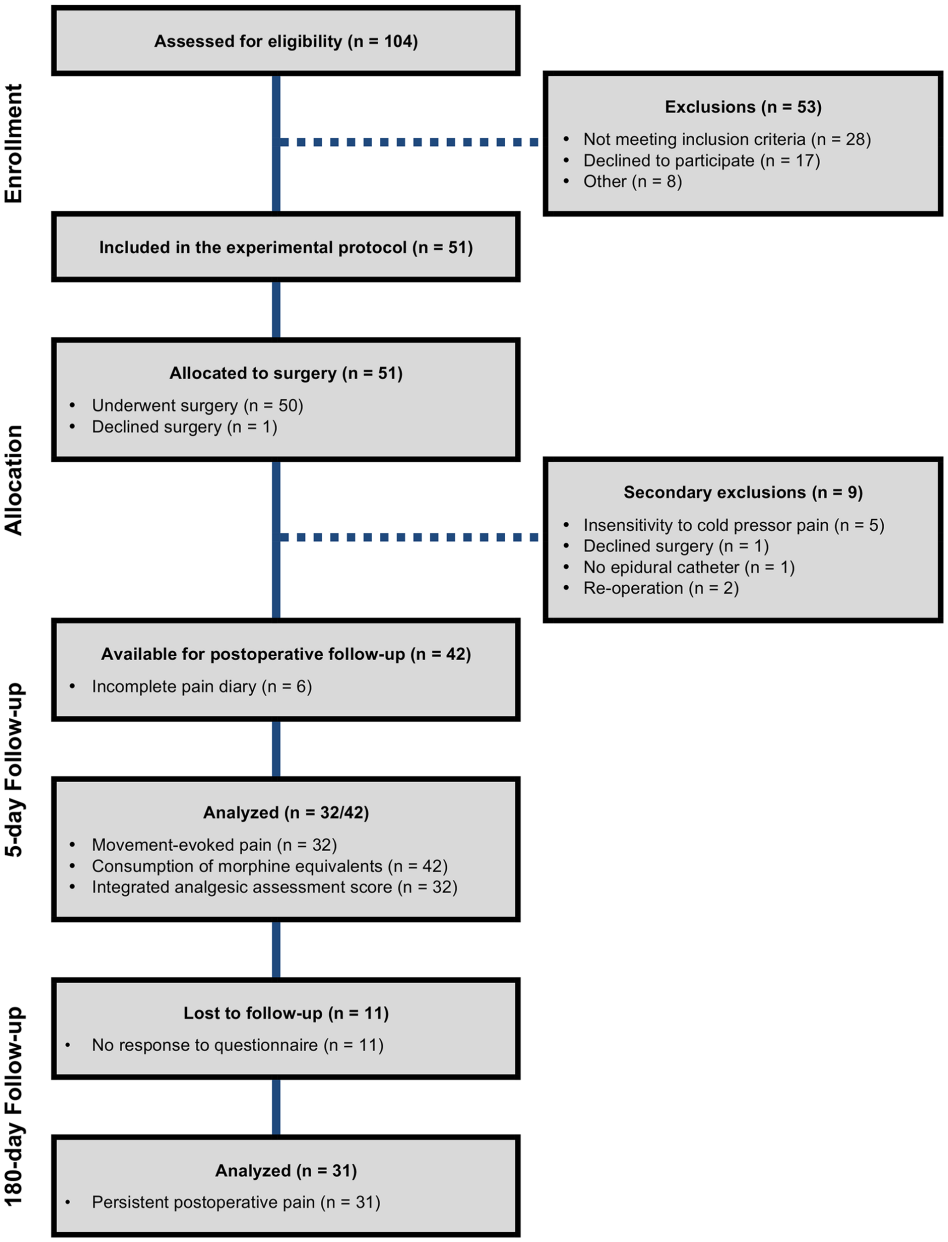
Flow chart. Illustration of the patient selection process, reasons for exclusion and number of patients analyzed for the primary and secondary outcomes.

We included 51 patients (50 males and 1 female) in the experimental protocol (Fig. 2). Preoperatively, all patients were pain-free and presented with a normal sensory function in the planned surgical area. Eight patients (7 males and 1 female) were subsequently excluded due to secondary exclusion criteria and one declined surgery. Accordingly, 42 male patients with a median age of 19 [17]–[23] years were available for postoperative follow-up. Patient demographic and clinical characteristics, preoperative predictors and primary and secondary postoperative pain-related outcomes are depicted in Table 1.

**Table 1:** Patient Characteristics, Preoperative Predictors and Postoperative Pain-related Outcomes (N = 42).

Variables	
**Patient characteristics**	
Age (years)	19 [17]–[23]
Height (cm)	184.1±7.6
Weight (kg)	70.4±11.5
Pectus excavation depth (cm)	5.4±1.4
**Preoperative Conditioned Pain Modulation**	
PPT 1 (kPa)	960±226
PPT 2 (kPa)	1109±334
CPM, absolute change (kPa)	150±245
CPM%, relative change (%)	17±30
CPTP (NRS 0–10)	9 [7]–[10]
CPTU (NRS 0–10)	9 [7]–[10]
Cold pain detection (s)	25 [14]–[31]
Cold pain tolerance (s)	120 [52–120]
**Preoperative Psychological Questionnaires**	
STAI score	37 [32]–[47]
BDI score	5 [3]–[12]
S-PCS score	16 [8]–[26]
S-PCS Helplessness score	7 [2]–[15]
S-PCS Rumination score	7 [4]–[13]
S-PCS Magnification score	1 [0–4]
**Anesthesia, Surgery and Epidural Analgesia**	
Duration of anesthesia (min)	109 [98–130]
Duration of surgery (min)	33 [25]–[39]
Intraoperative Fentanyl (μg)	200 [100–225]
Intraoperative Bupivacaine (mg)	15.7±8.2
Intraoperative Morphine (μg)	314±164
Postoperative Bupivacaine (POD 0–2) (mg) (n = 41)	654 [568–759]
Postoperative Morphine (POD 0–2) (mg) (n = 40)	5.8 [4.7–7.5]
**Primary Outcome (POD 180)**	
Persistent postoperative pain (of any degree) (n = 31)	15 (48%)
Daily pain	4 (13%)
Weekly pain	7 (23%)
More rarely pain	4 (13%)
Worst pain intensity (NRS 0–10)	3 [2]–[3]
Mild pain intensity (NRS 0–10)	0 [0–0]
Average pain intensity (NRS 0–10)	1 [0–1]
Current pain intensity (NRS 0–10)	0 [0–0]
**Secondary Postoperative Pain-related Outcomes (POD 2-5)**	
PAR (NRS 0–10) (n = 32)	4±2
MEP (NRS 0–10) (n = 32)	5±2
Morphine consumption (mg kg^−1^ day^−1^)	1.0±0.3
Integrated analgesic assessment score (n = 32)	0±79

Patient characteristics, preoperative predictors and postoperative pain-related outcomes in a study designed to assess whether preoperative conditioned pain modulation and situational pain catastrophizing can predict measures of clinical postoperative pain.

Data are presented as numbers (percentages), mean ± standard deviation or median [interquartile range] depending on distribution profile.

*n*  =  number of observations if *n* is different from total (N = 42); kPa  =  kilopascal; STAI  =  state part of the Spielberger's State-Trait Anxiety Inventory; BDI  =  Beck's Depression Inventory; S-PCS  =  Situational Pain Catastrophizing Scale administered in connection with cold pressor test; PPT 1 =  pressure pain threshold (test pain before cold pressor test); PPT 2 =  pressure pain threshold (test pain after 120 s cold pressor test); CPM  =  conditioned pain modulation (i.e., difference between PPT 1 and PPT 2); CPTP  =  worst (maximum) pain intensity associated with cold pressor test; CPTU  =  worst (maximum) unpleasantness associated with cold pressor test; Cold pain detection  =  time to pain detection during cold pressor test; Cold pain tolerance  =  total hand immersion time during cold pressor test; Persistent postoperative pain  =  pain assessed according to responses to the Brief Pain Inventory – Short Form (BPI) at six months (POD 180); POD  =  postoperative day; PAR  =  postoperative pain at rest; MEP  =  postoperative movement-evoked pain; NRS 0–10 = 11-point numerical rating scale; Integrated analgesic assessment score  =  a composite measure of movement-evoked pain intensity and morphine consumption: Pain intensity scores and morphine consumption for each patient were rank ordered, subtracted from the mean rank, expressed as a percentage difference from the mean rank and added together (i.e., pain rank score + morphine rank score) to yield and integrated analgesic assessment score (ranging from −200 to +200%; with the highest positive score indicating the most pain).

### Preoperative Predictor and Confounder Assessments and Crude Associations

There was a 17% increase in the mean pressure pain thresholds before and after cold pressor test (960±226 vs. 1109±334 kPa; *t* = −4.0, *P* = 0.0003) (Table 1). Change in the absolute pressure pain threshold values was 150±245 kPa. The S-PCS score was median 18 [8]–[26] with median subscale scores of 7 [2]–[15] for helplessness, 7 [4]–[13] for rumination, and 1 [0–4] for magnification. As regards the potential confounding variables, the STAI score was median 37 (range: 22–60) and the BDI score was median 5 (range: 0–30). Spearman's rank correlations for preoperative, perioperative, and postoperative predictors, confounders and outcomes are depicted in Table 2. Notably, there were no significant associations between CPM responses and measures related to the conditioning pain stimulation (i.e., worst cold pressor pain and unpleasantness scores and cold pressor pain detection and tolerance thresholds). Furthermore, there were no significant associations between CPM response, S-PCS, BDI and STAI scores. The BDI and STAI scores, the duration of surgery, the intraoperative use of fentanyl and epidural analgesia were not found to be significantly associated with any of the postoperative pain-related outcomes. We found no evidence of associations between experimental pain measures, such as test pain or conditioning pain, and any of the primary or secondary outcomes; however, unpleasantness of cold pressor pain was associated with both morphine consumption (Rho  = 0.31; *P* = 0.04) and the integrated analgesic assessment score (Rho  = 0.47; *P* = 0.007). We found no evidence of associations between measures of postoperative pain intensity and analgesic consumption. In other words, patients reporting postoperative pain of high intensity do not necessarily consume more morphine, and vice versa.

**Table 2:** Spearman's Rank Correlation Matrix for Preoperative, Perioperative, and Postoperative Study Variables Including Predictors, Confounders and Outcomes.

Variable	1	2	3	4	5	6	7	8	9	10	11	12	13	14	15	16	17	18
1. STAI score	-																	
2. BDI score	0.19	-																
3. PPT 1 (kPa)	0.02	0.16	-															
4. PPT 2 (kPa)	0.07	0.29	**0.71**	-														
5. Cold pain detection (s)	−0.03	−0.24	0.17	0.26	-													
6. Cold pain tolerance (s)	0.05	−0.17	**0.32**	0.28	**0.59**	-												
7. CPTP (NRS 0–10)	−0.14	0.20	−0.22	−0.25	**−0.53**	**−0.83**	-											
8. CPTU (NRS 0–10)	−0.21	0.20	−0.26	**−0.32**	**−0.49**	**−0.72**	**0.85**	-										
9. CPM (%) relative change (%)	0.16	0.21	0.01	**0.67**	0.27	0.09	−0.20	−0.22	-									
10. S-PCS score	0.14	0.30	−0.08	0.08	**−0.38**	**−0.46**	**0.50**	**0.46**	0.21	-								
11. Duration of surgery (min)	−0.10	−0.02	**0.40**	0.29	0.08	0.16	−0.25	−0.27	0.01	−0.27	-							
12. Epidural analgesia (mg)	−0.14	−0.14	0.22	0.00	0.09	0.25	−0.20	−0.19	−0.17	**−0.33**	**0.31**	-						
13. Fentanyl (μg)	−0.02	0.23	0.29	0.19	−0.13	−0.02	−0.18	−0.15	0.02	−0.06	**0.35**	0.18	-					
14. PAR (NRS 0–10)	0.23	0.30	0.22	0.29	−0.19	−0.07	0.13	0.20	0.22	**0.38**	0.14	−0.01	0.14	-				
15. MEP (NRS 0–10)	0.17	0.08	−0.16	−0.02	−0.30	−0.16	0.20	0.30	0.19	**0.40**	−0.22	−0.14	−0.13	**0.73**	-			
16. Morphine (mg kg^−1^ day^−1^)	−0.03	−0.03	0.03	−0.26	−0.19	−0.09	0.22	**0.31**	**−0.46**	−0.18	−0.15	−0.06	−0.13	0.06	−0.03	-		
17. Integrated analgesic score	0.20	0.19	−0.07	−0.19	−0.33	−0.17	0.34	**0.47**	−0.20	0.25	−0.29	−0.18	−0.15	**0.56**	**0.70**	**0.69**	-	
18. Pain at POD 180 (NRS 0–10)	0.07	0.04	0.29	0.20	0.03	−0.27	0.13	−0.02	−0.03	**0.54**	0.02	−0.06	0.11	0.23	0.39	−0.01	0.36	-

Spearman's rank correlation coefficients for all pairs of preoperative, perioperative, and postoperative study variables including predictors, confounders and outcomes. Total *n* for the pairwise correlations with pre- and intraoperative variables is 42. For postoperative pain-related outcomes any pairwise correlation has a total *n* of 32 with the exception of morphine (n = 42) and persistent postoperative pain at six months (POD 180) (n = 15). Coefficients with significance levels of 0.05 or less are printed in **bold**.

STAI  =  state part of the Spielberger's State Trait-Anxiety Inventory; BDI  =  Beck's Depression Inventory; PPT 1 =  pressure pain threshold (test pain before cold pressor test); PPT 2 =  pressure pain threshold (test pain after 120 s cold pressor test); kPa  =  kilopascal; Cold pain detection  =  time to pain detection during cold pressor test; Cold pain tolerance  =  total hand immersion time during cold pressor test; CPTP  =  worst pain intensity associated with cold pressor test; CPTU  =  worst unpleasantness associated with cold pressor test; NRS 0–10 = 11-point numerical rating scale; CPM  =  conditioned pain modulation (i.e., difference between PPT 1 and PPT 2); S-PCS  =  the Situational Pain Catastrophizing Scale administered in connection with cold pressor test; Epidural analgesia  =  total intraoperative dose of 0.25% bupivacaine + morphine, 50 µg ml^−1^; Fentanyl  =  total dose of intraoperative fentanyl; PAR  =  postoperative pain at rest (POD 2–5); MEP  =  postoperative movement-evoked pain (POD 2–5); Morphine  =  postoperative morphine consumption (POD 2–5); Integrated analgesic assessment score  =  a composite measure of movement-evoked pain intensity and morphine consumption: Pain intensity scores and morphine consumption for each patient were rank ordered, subtracted from the mean rank, expressed as a percentage difference from the mean rank and added together (i.e., pain rank score + morphine rank score) to yield and integrated analgesic assessment score (ranging anywhere from −200 to +200%; with the highest positive score indicating the most pain); Pain at POD 180 was assessed according to response to the question: “Please rate your pain by circling the one number that best describes your pain on the average” from the Brief Pain Inventory – Short Form (BPI). The BPI rates worst, average, current, and least pain using simple numeric rating scales (NRS) ranging from 0 to 10, where 0 =  no pain and 10 =  the worst pain imaginable.

### Primary Outcome – Persistent Postoperative Pain

Thirty-one patients (74%) responded to our questionnaire assessment at six months after surgery (Figure 2). Of these, 15 patients (48%) complained of persistent postoperative pain as a direct consequence of the surgical procedure (Table 2). Pain was present daily in four patients (13%), weekly in seven (23%) and more rarely in four (13%). Pain was most frequently located to the ribcage at level with the surgical incisions or behind the sternum. Pain was described as movement-evoked, e.g., heavy lifting and high intensity fitness training or associated with couching/sneezing/laughing or lying on the side. The reported average pain intensity score on NRS was less than 3 in all patients. Only one patient required intermittent use of analgesics (i.e., acetaminophen and morphine).

### Conditioned Pain Modulation and Persistent Postoperative Pain

The median CPM response in patients reporting pain at six months after surgery was 1.2% (95% CI: −7.4; 12.7%); in pain-free patients it was 9.0% (95% CI: −0.5; 23.7%). Accordingly, there was no significant difference between the two groups (*Z* = 0.99, *P* = 0.3).

### Situational Pain Catastrophizing and Persistent Postoperative Pain

The distributions of S-PCS scores were not significantly different for patients reporting pain at six months after surgery and pain-free patients, with median S-PCS scores of 11 (95% CI: 8; 24) and 15 (95% CI: 9; 24), respectively (*Z* = −0.04, *P* = 0.69).

### Secondary Postoperative Pain-related Outcomes

At postoperative days 2–5 mean level of movement-evoked pain was 5±2/10 and mean consumption of morphine equivalents was 1.0±0.3 mg kg^−1^ day^−1^; ranging from 30 to 110 mg/day (Table 1). There was no association between postoperative pain intensities and morphine consumption (Table 2). Four patients (12%) reported high pain while consuming higher-than-average morphine, whereas three patients (9%) reported low pain and consumed less–than-average morphine.

### *Conditioned Pain Modulation* and Secondary Postoperative Pain-related Outcomes

Parameter estimates with 95% confidence intervals from linear regression models of secondary postoperative pain-related outcomes are shown in Tables 3 to 5. A total of 22% of the variance in morphine consumption was explained by CPM (*P* = 0.001; Table 4, Model 1). In contrast, CPM was not related to movement-evoked pain intensity (*P* = 0.2; Table 3, Model 1) or the integrated analgesic assessment score (*P* = 0.3; Table 5, Model 1).

**Table 3:** Parameter Estimates from Regression Models of Postoperative Movement-evoked Pain Intensity (NRS 0–10) against Preoperative Conditioned Pain Modulation, Situational Pain Catastrophizing, Anxiety, and Depression.

	Model 1 (N = 32)			Model 2 (N = 31)			Model 3 (N = 31)		
Covariate	Parameter	Parameter estimate (95% CI)	*P* value	Parameter	Parameter estimate (95% CI)	*P* value	Parameter	Parameter estimate (95% CI)	*P* value
Constant	*α*	5.076 (4.294; 5.858)	**<0.001**	*α*	2.714 (0.886; 4.541)	**0.005**	*α*	2.705 (−0.189; 5.599)	0.066
*CPM%_i_*	*β*_1_	0.015 (−0.01; 0.04)	0.228	*β*_1_			*β*_1_		
Log[*S-PCS_i_*]	*β*_2_			*β*_2_	1.020 (0.352; 1.688)	**0.004**	*β*_2_	1.026 (0.298 1.753)	**0.007**
*STAI_i_*	*β*_3_			*β*_3_			*β*_3_	0.003 (−0.070; 0.076)	0.935
*BDI_i_*	*β*_4_			*β*_4_			*β*_4_	−0.017 (−0.121; 0.088)	0.744
Model fit									
F-statistic	F(1, 30) = 1.52, *P* = 0.23			F(1, 29) = 9.76, *P* = 0.004			F(3, 27) = 3.08, *P* = 0.04		
Adj. R- squared	0.02			0.23			0.17		

The effect of preoperative conditioned pain modulation and situational pain catastrophizing on patients' ratings of movement-evoked pain intensity following chest wall surgery. State anxiety and depression are solely included in the models to statistically control the effect of pain catastrophizing on postoperative pain (a priori confounders). The regression outputs indicate that situational pain catastrophizing significantly predicted postoperative movement-evoked pain intensity, per se (**Model 2**), and independently of anxiety and depression (**Model 3**). The adjusted R-squared values indicate that up to 23% of variance in the dependent variable (postoperative movement-evoked pain) can be explained by the independent variable Log[S-PCS]. Conditioned pain modulation was not related with postoperative movement-evoked pain (**Model 1**).

N  =  the number of observations used in the regression analysis; 95% CI  = 95% confidence interval for the coefficients; *P* value  =  two-tailed *P* values used in testing the null hypothesis that the coefficient (parameter) is 0 using an alpha of 0.05; CPM%  =  conditioned pain modulation (i.e., relative difference between pressure pain thresholds obtained before and after 120 s cold pressor test); Log[S-PCS]  =  Situational Pain Catastrophizing Scale score (log-transformed); STAI  =  Spielberger's State Anxiety and Inventory score; BDI  =  Beck's Depression Inventory score; F-statistic  =  the mean square model divided by the mean square residual. The *P* value associated with the F-statistic is used in testing the null hypothesis that all of the model coefficients are 0; Adj. R-squared  =  a modified version of R-squared that has been adjusted for the number of predictors in the model.

**Table 4:** Parameter Estimates from Regression Models of Postoperative Consumption of Morphine Equivalents (mg/kg/day) against Conditioned Pain Modulation, Situational Pain Catastrophizing, Anxiety, and Depression.

	Model 1 (N = 42)			Model 2 (N = 41)			Model 3 (N = 41)		
Covariate	Parameter	Parameter estimate (95% CI)	*P* value	Parameter	Parameter estimate (95% CI)	*P* value	Parameter	Parameter estimate (95% CI)	*P* value
Constant	*α*	1.114 (1.028; 1.200)	**<0.001**	*α*	1.211 (0.978; 1.444)	**<0.001**	*α*	1.109 (0.715; 1.503)	**<0.001**
*CPM%_i_*	*β*_1_	−0.005 (−0.007; −0.002)	**0.001**	*β*_1_			*β*_1_		
Log[*S-PCS_i_*]	*β*_2_			*β*_2_	−0.064 (−0.149; 0.020)	0.132	*β*_2_	−0.072 (−0.162; 0.018)	0.115
*STAI_i_*	*β*_3_			*β*_3_			*β*_3_	0.003 (−0.007; 0.013)	0.537
*BDI_i_*	*β*_4_			*β*_4_			*β*_4_	0.001 (−0.013; 0.015)	0.924
Model fit									
F-statistic	F(1, 40) = 12.48, *P* = 0.001			F(1, 39) = 2.36, P = 0.13			F(3, 37) = 0.91, *P* = 0.44		
Adj. R- squared	0.22			0.03			−0.07		

The effect of preoperative conditioned pain modulation and situational pain catastrophizing on patients' consumption of morphine equivalents following chest wall surgery. State anxiety and depression are solely included in the models to statistically control the effect of pain catastrophizing on postoperative morphine consumption (a priori confounders). The regression outputs indicate that conditioned pain modulation significantly predicted postoperative morphine consumption (**Model 1**). Situational pain catastrophizing was not related with postoperative movement-evoked pain (**Models 2 and 3**).

N  =  the number of observations used in the regression analysis; 95% CI  = 95% confidence interval for the coefficients; P value  =  two-tailed P values used in testing the null hypothesis that the coefficient (parameter) is 0 using an alpha of 0.05; CPM%  =  conditioned pain modulation (i.e., relative difference between pressure pain thresholds obtained before and after 120 s cold pressor test); Log[S-PCS]  =  Situational Pain Catastrophizing Scale score (log-transformed); STAI  =  Spielberger's State Anxiety and Inventory score; BDI  =  Beck's Depression Inventory score; F-statistic  =  the mean square model divided by the mean square residual. The P value associated with the F-statistic is used in testing the null hypothesis that all of the model coefficients are 0; Adj. R-squared  =  a modified version of R-squared that has been adjusted for the number of predictors in the model.

**Table 5:** Parameter Estimates from Regression Models of an Integrated Analgesic Assessment Score based on Morphine Consumption and Movement-evoked Pain Intensity Scores (−200 to +200%) against Conditioned Pain Modulation, Situational Pain Catastrophizing, Anxiety, and Depression.

	Model 1 (N = 32)			Model 2 (N = 31)			Model 3 (N = 31)		
Covariate	Parameter	Parameter estimate (95% CI)	*P* value	Parameter	Parameter estimate (95% CI)	*P* value	Parameter	Parameter estimate (95% CI)	*P* value
Constant	*α*	7.324 (−24.57; 39.22)	0.642	*α*	−55.051 (−135.7; 25.6)	0.173	*α*	−92.944 (−218.9; 33.0)	0.142
*CPM%_i_*	*β*_1_	−0.514 (−1.52; 0.50)	0.307	*β*_1_			*β*_1_		
Log[*S-PCS_i_*]	*β*_2_			*β*_2_	22.671 (−6.8; 52.1)	0.126	*β*_2_	18.502 (−13.2; 50.2)	0.241
*STAI_i_*	*β*_3_			*β*_3_			*β*_3_	1.089 (−2.1; 4.3)	0.489
*BDI_i_*	*β*_4_			*β*_4_			*β*_4_	0.629 (−3.9; 5.2)	0.778
Model fit									
F-statistic	F(1, 30) = 1.08, *P* = 0.31			F(1, 29) = 2.48, *P* = 0.13			F(3, 27) = 1.08, *P* = 0.38		
Adj. R- squared	0.03			0.05			0.08		

The effect of preoperative conditioned pain modulation and situational pain catastrophizing on an integrated analgesic assessment score based on morphine consumption and movement-evoked pain intensity scores following chest wall surgery. State anxiety and depression are solely included in the models to statistically control the effect of pain catastrophizing on the integrated analgesic assessment score (a priori confounders). The regression outputs indicate that conditioned pain modulation and situational pain catastrophizing are not significantly related with the integrated analgesic assessment score (**Models 1–3**).

N  =  the number of observations used in the regression analysis; 95% CI  = 95% confidence interval for the coefficients; P value  =  two-tailed P values used in testing the null hypothesis that the coefficient (parameter) is 0 using an alpha of 0.05; CPM%  =  conditioned pain modulation (i.e., relative difference between pressure pain thresholds obtained before and after 120 s cold pressor test); Log[S-PCS]  =  Situational Pain Catastrophizing Scale score (log-transformed); STAI  =  Spielberger's State Anxiety and Inventory score; BDI  =  Beck's Depression Inventory score; F-statistic  =  the mean square model divided by the mean square residual. The P value associated with the F-statistic is used in testing the null hypothesis that all of the model coefficients are 0; Adj. R-squared  =  a modified version of R-squared that has been adjusted for the number of predictors in the model.

### *Situational Pain Catastrophizing* and Secondary Postoperative Pain-related Outcomes

Situational pain catastrophizing explained 23% of the variation in postoperative movement-evoked pain intensity (*P* = 0.004; Table 3, Model 2). The significant association between situational pain catastrophizing and pain intensity remained when statistically adjusted for anxiety and depression (*P* = 0.007; Table 3, Model 3). In contrast, situational pain catastrophizing was not related to morphine consumption (*P* = 0.1; Table 4, Model 2) or the integrated analgesic assessment score (*P* = 0.1; Table 5, Model 2), and remained unrelated when statistically adjusted for anxiety and depression (*P* = 0.1; Table 4, Model 3 and *P* = 0.2; Table 5, Model 3).

### Interpreting and Visualizing the Regression Models of Secondary Postoperative Pain-related Outcomes

**Figure 3:**
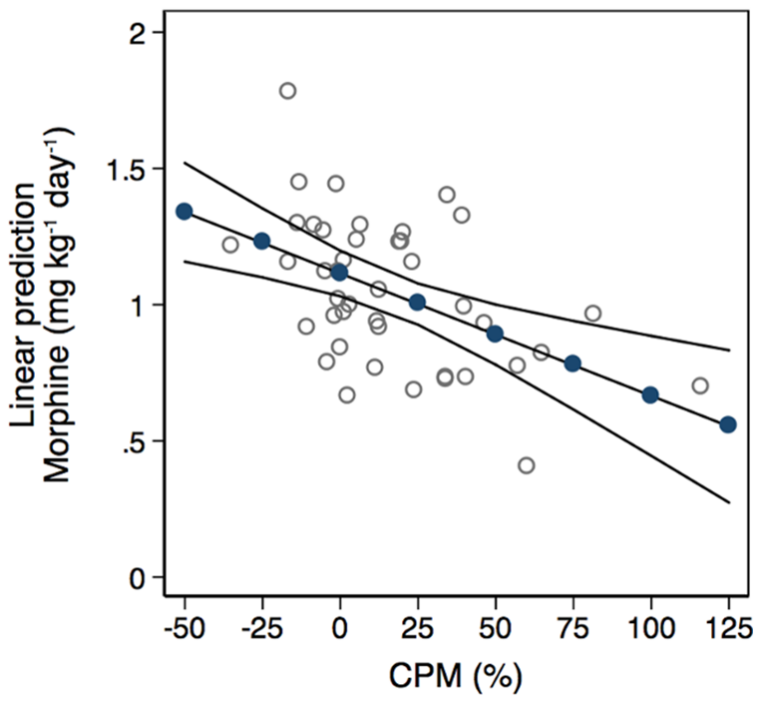
Preoperative situational pain catastrophizing and postoperative movement-evoked pain. Adjusted means (filled circles) with 95% confidence intervals (continuous solid lines) of the patient's postoperative movement-evoked pain intensity (NRS 0–10) given different log-transformed values of preoperative situational pain catastrophizing (log[S-PCS]), adjusted for anxiety and depression by averaging across the values of the state part of the Spielberger's State-Trait Anxiety Inventory (STAI) and Beck's Depression Inventory (BDI) (average marginal values). The hollow circles represent overlaid scatterplots of log[S-PCS] versus postoperative movement-evoked pain intensity.

**Figure 4:**
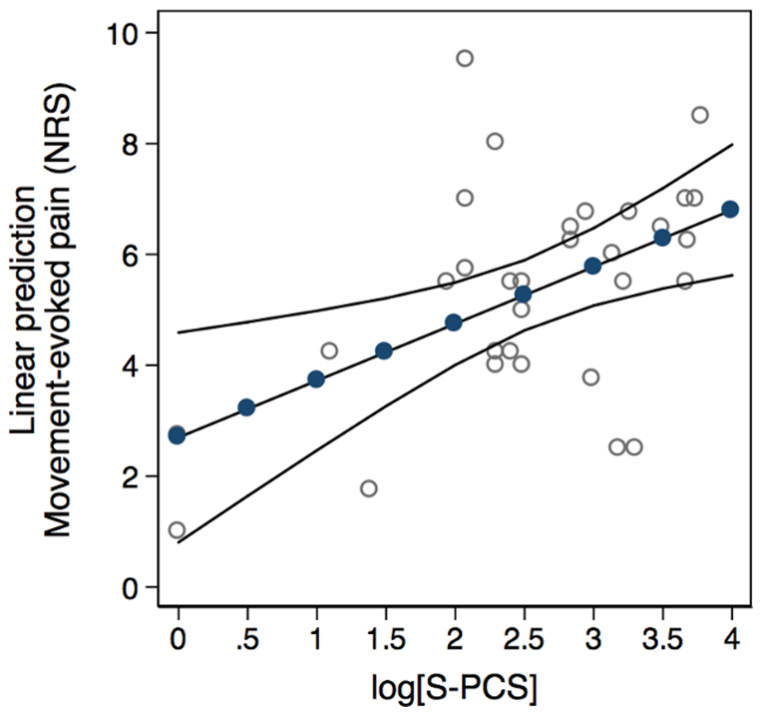
Preoperative conditioned pain modulation and postoperative morphine consumption. Adjusted means (filled circles) with 95% confidence intervals (continuous solid lines) of the patient's postoperative morphine consumption given different values of preoperative conditioned pain modulation (CPM %). The hollow circles represent overlaid scatterplots of CPM % versus postoperative morphine consumption.

The above-mentioned regressions could be used to compute the predicted mean for the patient's postoperative movement-evoked pain intensity and morphine consumption for any given level of his preoperative CPM response or situational pain-related catastrophic thinking. By using the multiple regression model considering the patient's preoperative level of situational pain catastrophizing, anxiety and depression (Table 3, Model 3), a preoperative PCS score of e.g. 16 (i.e., 2.8 on log-scale) would compute the mean of the patient's postoperative movement-evoked pain intensity to 5.5 (95% CI: 4.9–6.2) (Figure 3). In comparison, patients with low and high levels of catastrophic thinking (e.g. equal to the 25^th^ and 75^th^ percentiles of the S-PCS score) would yield predicted means at 4.8 (95% CI: 4.1; 5.5) and 6.0 (95% CI: 5.2; 6.8), respectively. Similarly, a CPM response of e.g. 25% would yield a predicted mean postoperative morphine consumption of 1.0 (95% CI: 0.9; 1.1) mg kg^−1^ day^−1^ using the simple regression model in which patients' morphine consumption was uniquely predicted from their preoperative CPM response (Figure 4 and Table 4, Model 1). This latter calculation corresponds to a predicted postoperative morphine consumption of 70 (95% CI: 65; 76) mg/day for the average patient with a body weight of 70 kg. If the patient had an inefficient pain inhibition capacity equal to the lower quartile of the CPM score, the predicted morphine consumption would be estimated at 79 (95% CI: 73; 85) mg/day.

## Discussion

The present study assessed whether preoperative experimental pain modulation can predict measures of clinical postoperative pain. This was investigated in a cohort of consecutive healthy pain-free male adolescents undergoing a standardized thoracic surgical procedure using uniform anesthesia and analgesia protocols. The hypothesis that less efficient endogenous pain inhibition capacity would increase the risk of developing persistent postoperative pain was not supported by our data. However, our data indicated that patients with higher levels of preoperative situational catastrophic thinking reported higher movement-evoked pain intensity scores in the acute postoperative phase. Furthermore, consumption of morphine equivalents in the acute setting depended on preoperative CPM; thus, patients with less efficient CPM (negative CPM scores) consumed more morphine than patients with efficient CPM (positive CPM scores), and vice versa.

### Prediction of Persistent Postoperative Pain

Contrary to some previous studies [14], [59], the current study did not prove preoperative CPM efficiency predictive of persistent postoperative pain development. Similarly, the lack of predictive value of situational pain catastrophizing does not add to the moderate evidence that preoperative measures of anxiety and pain catastrophizing play a role in the development of persistent postoperative pain [60]. However, our results are in agreement with other studies showing a non-significant association between the total Pain Catastrophizing Scale score and persistent postoperative pain [57], [61]–[63]. Major differences between study populations (preoperative pain, age, underlying disease and type of surgery) may explain the diverging results. However, another probable explanation for our findings may be attributed to the decreased prevalence of clinically relevant persistent postoperative pain in our study population at six months. The possible bias of these responses should be considered as they may have caused a lack of satisfactory differentiation between clinically relevant pain and discomfort. Although we used the BPI to evaluate the degree to which pain influenced a number of daily activities, it did not facilitate persistent pain status classification. We cannot rule out that such information bias could have led to outcome misclassification and potential attenuation of risk estimates. Along this line it is possible to hypothesize that pain perception may be reported differently in an otherwise healthy young population where low intensity pain may be tacitly accepted in favor of an improved cosmetic appearance. Several questions remain unanswered and there is a need for further progress in determining the exact role of CPM and situational pain catastrophizing in the transition from acute to chronic (postoperative) pain. Additionally, it should be noted that other clinically relevant mechanisms may be important in persistent postoperative pain development.

### Prediction of Secondary Postoperative Pain-related Outcomes

#### Subjective Movement-evoked Pain Intensity

Our findings extend previous observations [17]–[20], [64]–[73] by showing that pain catastrophizing in response to experimental cold pressor pain explains a high amount of the variance in self-reported maneuver-specific postoperative pain intensity. Our results corroborate those of Strulov *et al.* showing that pain catastrophizing in response to experimental heat pain accounted significantly for the variance in cesarean section pain [20]. Moreover, the present findings coincide with previous studies showing that preoperative pain catastrophizing predicts acute postoperative pain measures independent of other psychological factors, including anxiety [17], [65], [68]–[71], depression [65], [68], [69], optimism [71], [72], and self-efficacy [71]. Contrary to the conclusions of a recent review that identified anxiety as one of the four most significant predictors for postoperative pain intensity [74], we did not find preoperative state anxiety to be an independent significant predictor of pain. Conversely, our findings may contribute to the few studies of the relationship between pain catastrophizing and anxiety suggesting that both cognitive and emotional factors must be assessed in postoperative pain management [17], [70], [72]. The proposed mechanisms by which pain catastrophizing negatively effects the experience of pain range from interpersonal to neurophysiological, including appraisal processes, attentional bias, behavioral coping, CNS mechanisms, and physiological responses to pain [16]. Accordingly, pain catastrophizing has been viewed both as an appraisal process in which painful stimuli are appraised in a primary (magnification, rumination) and secondary (helplessness) fashion and as a behavioral coping strategy employed by individuals experiencing pain to solicit supportive responses from others [16]. Furthermore, pain catastrophizing has been associated with a heightened attentional bias to the negative affective dimensions of pain-relevant stimuli and an inability to disengage from pain or pain cues, with altered CNS processes, and with activation in brain regions implicated in processing of affective dimensions of pain [16]. Thus, it has previously been hypothesized that pain catastrophizing may negatively influence CPM through multiple systems of complex anatomical circuitry that link cortical responses to pain with top-down processes that modulate pain [10], [28], [75]._ENREF_10 A few experimental studies in healthy volunteers have shown that individuals with high levels of catastrophizing demonstrate higher pain intensities and lower CPM effects [28], [31]. This suggests that the heightened pain reported by individuals higher in pain catastrophizing may be related to a disruption in the endogenous modulation of pain. However, as argued by some authors, it could also be that less efficient CPM systems may enhance the intensity of a painful experience, which in turn could cause higher catastrophizing [28]. In line with other studies [27], [29], we found no association between CPM response and catastrophizing. This indicates that pain catastrophizing is an independent psychological predictor of increased acute postoperative pain. However, the various conditions in which catastrophizing may function as a variable response to pain and pain modulation are sparsely investigated, hence we caution against drawing firm conclusions at this point. Collectively, our findings provide support for the use of psychological constructs in predicting postoperative pain and particularly for the clinical utility of assessing situational pain catastrophizing. This may be an important issue for future research as a shift from curative to preventive postoperative pain management could lead to substantial cost savings. Despite achievable preoperative identification of patients with a high level of catastrophic thinking, most clinical interventions (e.g., cognitive behavioral techniques) targeting pain catastrophizing remain unproven in the surgical setting.

#### Consumption of Morphine Equivalents

To date, very few studies have assessed the CPM effect as predictor of analgesic efficacy and results diverge [76]. Despite our small sample, we were able to obtain clinically applicable and statistically significant effect estimates of postoperative morphine consumption. This finding may have important implications for the development of tailored analgesic drug administration in future surgical patients. Although we did not assess CPM postoperatively, it may be hypothesized that morphine enhanced postoperative pain inhibition capacity resulting in reduced morphine requirements. This is supported by recent studies showing that opioid analgesics potentiated the effect of descending pain inhibition in healthy volunteers [77] and in neuropathic pain patients [78]. We did, however, not successfully establish an association between CPM and postoperative pain intensity, and it may thus be speculated that this is a mere consequence of increased morphine consumption. In other words, it is plausible that patients with deficient preoperative pain inhibition capacity experienced higher postoperative pain levels creating a need for a higher morphine dose to maintain an acceptable state of comfort compared to patients with more efficient CPM. The mechanisms by which psychological factors interfere with the response to analgesics remain unclear. In agreement with the present findings, previous studies have reported that levels of pain catastrophizing are not associated with postoperative analgesic consumption [17], [18], [20], [67]. In contrast, other studies have found higher levels of catastrophizing associated with poorer effectiveness of analgesic treatment of experimental and clinical pain [64], [69], [79]–[81]. We believe the explanation to this is rooted in differences in study design and methodology.

#### Integrated Analgesic Assessment Score

Preoperative CPM and situational pain catastrophizing were not predictive of the proposed integrated analgesic assessment score. One possible explanation for this may be attributed to our co-administration of several analgesics. Despite the use of a standardized analgesic regimen, we cannot rule out that the administration of several analgesics may have resulted in differential synergistic effects across patients. Another explanation for this observation may be based on evidence that a combined endpoint may cause problems if the predictor effect is in opposite directions for different outcomes included in the composite endpoint [82]. However, it is worth mentioning that normalizing data on a per-subject basis enabled identification of patients with differential treatment effects on pain and morphine consumption outcome and that such an approach may facilitate comparison of similar studies in the future. Though there is little reason to suspect that the proposed integrated index would not measure therapeutic efficacy at least as well as the individual components, future studies may wish to comprehensively assess the validity of this method [54].

### Limitations

Some of the limitations of the present study have implications for the generalizability of findings. Although we used a highly standardized surgical procedure and protocols for anesthesia and analgesia in a homogenous study population, inevitable differences in anesthesia, analgesia and surgically induced tissue injury and inflammation may have affected postoperative pain perception differently across patients. However, we found no evidence of significant associations between measures related to the anesthesia, analgesia, and complexity of surgery and study outcomes. Furthermore, patients in this study were primarily pain-free male adolescents who were not suffering from chronic pain before undergoing surgery. The applicability of the present findings to patients suffering from pain-related conditions thus remains uncertain. We acknowledge that our results are based on a small sample and that results need to be confirmed and replicated in larger studies. However, the homogeneous patient sample free from the usual bias related to pain, social and economical parameters, the uniform surgical procedure and the fact that all patients were operated on by the same surgeon and investigated by the same investigator strengthen our findings.

### Conclusions

In conclusion, preoperative CPM scores and levels of pain catastrophizing in response to experimental pain were not predictive of persistent postoperative pain development. Analyses of secondary pain-related outcomes, however, indicated that reduced pain-inhibition may contribute to enhanced clinical postoperative pain and morphine consumption. If replicated and confirmed in larger samples, this may potentially enable clinicians to tailor individualized analgesic drug administration and thus hypothetically improve postoperative pain management in future patients.
